# Gendered medicinal plant knowledge contributions to adaptive capacity and health sovereignty in Amazonia

**DOI:** 10.1007/s13280-016-0826-1

**Published:** 2016-11-22

**Authors:** Isabel Díaz-Reviriego, Álvaro Fernández-Llamazares, Matthieu Salpeteur, Patricia L. Howard, Victoria Reyes-García

**Affiliations:** 1 Institut de Ciència i Tecnologia Ambientals (ICTA), Universitat Autònoma de Barcelona, Barcelona, Spain; 2 Internet Interdisciplinary Institute (IN3), Universitat Oberta de Catalunya, Barcelona, Spain; 3 Metapopulation Research Centre (MRC), Department of Biosciences, University of Helsinki, Helsinki, Finland; 4 Centre d’Écologie Fonctionnelle et Évolutive (CEFE), CNRS UMR 5175, Montpellier, France; 5 Department Social Sciences, Wageningen University, Wageningen, The Netherlands; 6 Center for Biocultural Diversity Studies, School of Anthropology and Conservation, University of Kent, Canterbury, UK; 7 Institució Catalana de Recerca i Estudis Avançats (ICREA), Barcelona, Spain

**Keywords:** Gendered knowledge, Knowledge diversity, Knowledge redundancy, Local knowledge systems, Local medical systems, Tsimane’

## Abstract

Local medical systems are key elements of social-ecological systems as they provide culturally appropriate and locally accessible health care options, especially for populations with scarce access to biomedicine. The adaptive capacity of local medical systems generally rests on two pillars: species diversity and a robust local knowledge system, both threatened by local and global environmental change. We first present a conceptual framework to guide the assessment of knowledge diversity and redundancy in local medicinal knowledge systems through a gender lens. Then, we apply this conceptual framework to our research on the local medicinal plant knowledge of the Tsimane’ Amerindians. Our results suggest that Tsimane’ medicinal plant knowledge is gendered and that the frequency of reported ailments and the redundancy of knowledge used to treat them are positively associated. We discuss the implications of knowledge diversity and redundancy for local knowledge systems’ adaptive capacity, resilience, and health sovereignty.

**Electronic supplementary material:**

The online version of this article (doi:10.1007/s13280-016-0826-1) contains supplementary material, which is available to authorized users.

## Introduction

Local medical systems involve the knowledge, beliefs, and behaviors relating to health and illness, local natural resources attributed with medicinal properties, and social actors and groups living in small-scale populations (Kleinman 1978, 1980). Local medical systems are critical to the resilience and adaptive capacity of many indigenous peoples and rural communities with scarce access to biomedicine. ‘Resilience’ refers to “the capacity of a social-ecological system to absorb recurrent disturbances so as to retain essential structures, processes and feedbacks” (Adger et al. 2005, p. 1036), whereas ‘adaptive capacity’ refers to “the preconditions necessary to enable adaptation, including social and physical elements, and the ability to mobilize these elements” (Nelson et al. 2007, p. 397). Local medical systems contribute to resilience and adaptive capacity because they provide culturally appropriate, locally accessible, and affordable health care options (Kassam et al. 2010; Santoro et al. 2015).

Local medical systems generally rely on two main pillars: plant species diversity and local knowledge. Species diversity is related to the locally available spectrum of chemical components used for medicinal purposes, where the higher the diversity of available plant species, the greater the potential range of illnesses that can be treated. Species diversity also relates positively to functional redundancy, that is, the fact that more than one species can be used to treat a given illness. Redundancy is thus a key attribute of resilient local medical systems, given that, if a particular species becomes locally unavailable, others may substitute (Alburquerque and Oliveira 2007; Santoro et al. 2015). The second pillar of local medical systems is a complex and dynamic local knowledge system. Local knowledge systems are generally threatened and eroding but, at the same time, many are also dynamic and adaptive (Reyes-García 2015). People’s ability to generate, transform, accommodate, transmit, and apply local knowledge in an integrative way is critical to the resilience of such systems (Ellen et al. 2005; Mathez-Stiefel et al. 2012), and the erosion of medicinal plant knowledge may compromise a community’s adaptive capacity and resilience more generally. In this paper, we focus on this second pillar of local medical systems.

Local knowledge is unevenly distributed (Bruschi et al. 2011; Guimbo et al. 2011), which means that it is diverse. Knowledge diversity partly arises from specialization, which is related to social relations and identities. For example, shamans, herbalists, and midwives may hold different corpora of medicinal knowledge and their knowledge also differs from lay knowledge. Knowledge also varies according to differential access to ecosystems and landscapes (e.g., forests) and therefore to different species and the knowledge about these species, interests in medicinal plants and healing, and to the health concerns of the people treated (e.g., elderly, infants). Furthermore, there is a gender division of labor and responsibilities around care giving and healing that also generally leads to different behaviors and knowledge (e.g., Rocheleau et al. 1996; Pfeiffer and Butz 2005). Indeed, differences in the knowledge held by women and men, or gendered knowledge, are considered to be one of the most significant sources of intra-cultural knowledge variation (Howard 2003). Gender also intersects with other social divisions (Banerjee and Bell 2007), so that, for example, lay women belonging to different clan groups may hold distinct knowledge not only compared with men, but also with other women (see Boster et al. 1986).

One of the arguments that we make here is that attention to gendered knowledge is required if we are to understand how global environmental change (GEC) is affecting local medicinal knowledge systems and the resilience of local medical systems more generally. Local medical systems are particularly vulnerable to the impacts of GEC as changes in the vegetation and floristic composition of ecosystems directly influences the availability and use of medicinal plants (Hanazaki et al. 2013). Social change can also lead to changes in local medicinal knowledge systems, for example, through outmigration, formal education, and the increasing use of formal medicine (Byg et al. 2010; Giovannini et al. 2011), all of which are also likely to involve men and women differently.

Previous research analyzing the effects of GEC on local medical systems has focused on the challenges and vulnerabilities facing such systems and attributes that may contribute to their resilience. However, such research has tended to focus on the knowledge and practices of male specialists (i.e., shamans, healers) or experts (Kothari 2003; Gold and Clapp 2011). As a consequence, women’s medicinal knowledge and access to different medicinal resources is often neglected, despite the fact that lay women treat many common health concerns at household level (Wayland 2001; Finerman and Sackett 2003). We argue that recognizing the gendered nature of knowledge and understanding its dynamics provides insights into local vulnerability and adaptation to GEC. Since the challenges and vulnerabilities posed by GEC are not gender neutral (Denton 2002; Jost et al. 2016), specific attention must be given to the impacts on women’s knowledge and access to plant resources.

The aim of this paper is twofold. We propose a conceptual framework and initially test a new concept that can be applied to assessing the degree to which local medicinal knowledge systems may be adaptive and resilient to GEC and to other forms of social change that are affecting local medical systems and hence community resilience. The concept proposed, ‘functional knowledge redundancy,’ considers the importance of knowledge diversity within local medical systems. We argue that gendered knowledge diversity in particular may increase functional knowledge redundancy, and hence adaptive capacity and resilience in local medicinal knowledge systems. We partially test how this framework operates in a particular empirical situation using the case of medicinal plant knowledge among lay Tsimane’ of Bolivian Amazonia.

## Conceptual framework

### Knowledge diversity in knowledge systems

Researchers have long documented the fact that knowledge is unequally distributed within communities and that knowledge distribution is patterned. For example, Boster (1985, 1986) showed that, among the Aguaruna Amerindians of Peru, the distribution of knowledge about plant names follows a single, culturally shared model where knowledge is learned by different people to varying degrees. Knowledge distribution in Aguaruna society reflects Aguaruna social structure, which determines the importance of particular plants to particular people within it, where the variation between informants can be explained by factors that reflect differential knowledge: age, sex roles, and opportunities to learn (Boster 1985).

Knowledge diversity in local medical systems may arise as a result of a division of labor, or specialization, in different healing roles (e.g., shamans, healers, midwives, lay people) (see e.g., Lambert 2012) that potentially affect the performance of local medicinal knowledge systems. For example, in local medical systems, shamans and lay people play different roles (or functions) associated with different fields of action. Shamans may be responsible for treating spiritual illnesses and hold the related knowledge, while lay people may deal with mild afflictions.

Despite the fact that researchers have been aware of knowledge variation for many decades, they have rarely discussed the implications of knowledge diversity, or the differences in the knowledge held by different people, for the continuity of knowledge systems. Only recently have some researchers begun to argue that differentiated knowledge may be an asset for adaptation to changing social and ecological conditions. Ruelle and Kassam (2011), for example, studied plant knowledge among Standing Rock Nation elders in the United States. They found that elders hold knowledge about different plants and also different knowledge about the same plants. Differences in knowledge are partly linked to variations in species use, spatial distribution, and harvesting strategies. The authors conclude that such knowledge diversity allows the system to store more potential options for coping with change, which may improve the adaptive capacity of knowledge systems and therefore communities’ resilience.

### Knowledge redundancy and knowledge system adaptive capacity

The concept of *ecological redundancy* relates to the fact that more than one species can perform the same function or functions in a given ecosystem (Walker 1992). So, if one species disappears, functionally redundant species provide the system with the capacity to either resist change or bounce back after disturbance (resilience). Drawing on this concept, Albquerque and Oliveira (2007) proposed a model of utilitarian redundancy in local medical systems. They argue that such redundancy is important because it may reduce the potential impacts of overharvesting of particular species, allowing the functions of the system to be maintained even when a species population is reduced, thus ensuring local medical system resilience. The measure of redundancy that they propose aims to identify and quantify the relation between species richness (or medicinal plants species that are known) and their functions within the local medical system (uses or therapeutic categories according to the local illness classification system). This model assumes that, although people have preferences for some plants over others when treating any given ailment, other species can substitute when the preferred species are unavailable. The assumption dovetails with research that reports that people use substitute species only when preferred species are absent (Santoro et al. 2015). However, other research shows that, in such circumstances, people may instead recur to biomedical resources (Ferreira Junior et al. 2011).

Following Albuquerque and Oliveira’s utilitarian redundancy model (2007), we add a new concept—*functional knowledge redundancy*—which explicitly considers knowledge distribution within a local medical system. We define this as the number of species that each distinct group of social actors (population sub-group) knows that treat the same ailment. Such redundancy arises when different plant species are known by different groups of people to treat the same illness. Functional knowledge redundancy arises from any or all of those factors that account for intra-community knowledge diversity discussed above. The concept of functional knowledge redundancy serves to assess the overall capacity of a community (which, as per Boster 1985; Boster et al. 1986, is essentially the sum of knowledge held by all sub-population groups) to use different medicinal plants to treat health afflictions within a local medical system.

Overall, in local medical systems, knowledge diversity may affect the system’s performance. Functional knowledge redundancy may affect local medical system adaptive capacity by providing more options to treat ailments within a community. Knowledge diversity in local medical systems is likely to be expressed in relation to specialized and lay knowledge. Furthermore, within lay knowledge, gendered knowledge may increase functional knowledge redundancy, which helps communities to cope with a changing environment and may affect the adaptive capacity of the knowledge system and the resilience of the local medical system more generally.

## Case study

We worked in villages within the Tsimane’ Territory in the Department of Beni, Bolivian Amazonia. The majority of contemporary Tsimane’ are forager-horticulturalists who rely upon forest resources for food and medicine. The Tsimane’ succeeded in resisting Catholic and Protestant proselytism throughout the 1950s when the Protestant New Tribes Mission was established in the area. The Mission has profoundly influenced Tsimane’ culture through schooling and the provision of basic biomedical services. Contact with merchants, loggers, anthropologists, and conservationists have also affected Tsimane’ culture in diverse ways (Reyes-García et al. 2014). Even though the government has expanded primary health care services, access to biomedical healthcare is still very limited for the Tsimane’, especially for those living far from towns.

The Tsimane’ increasingly participate in the regional economy, mostly through direct sales of thatch palm, rice, and plantain in towns or to traders who visit their villages. Some Tsimane’ men also engage in wage labor for local loggers and ranchers. Tsimane’ livelihoods and healthcare are still highly self-sufficient and dependent on local forest resources, which increase their vulnerability to rapid ecosystem change (Fernández-Llamazares et al. 2015). For example, research has documented increased landscape fragmentation and deforestation (Paneque-Gálvez et al. 2013), processes that have direct consequences for the diversity and abundance of wildlife and therefore that affect Tsimane’ food security and health (Luz 2013; Zycherman 2013).

Rapid cultural changes have also been occurring among the Tsimane’ over the past few decades, which reverberate in their local medical system. For example, traditionally, the *cocojsi’* (shaman) played a very important medicinal role partly due to their ability to mediate between humans and spiritual beings that have curative powers. Today, only a few elders know how to administer traditional remedies against witchcraft, and people sometimes refer to them as *cocojsi’* (Huanca 2014), but they are not recognized generally as shamans. These practices persist mostly in more remote villages where people attribute illness and death to witchcraft. Similar changes are also observed in lay medicinal knowledge systems. The Tsimane’ have a great deal of local medicinal knowledge and, in general, lay Tsimane’ use medicinal plants to treat illnesses (Reyes-García et al. 2003). This lay knowledge seems to have positive effects on a household’s health profile (McDade et al. 2007). However, medicinal knowledge is changing as the Tsimane’ adapt to economic, social, political, and environmental change, where knowledge loss is more acute for men than for women, and for people living in villages that are closer to towns compared with those in remote villages (Reyes-García et al. 2013). Recent research has shown that the Tsimane’ use a combination of medicinal plants and pharmaceuticals to treat illnesses, presenting another example of medical pluralism (Calvet-Mir et al. 2008; Tanner and Rosinger 2014).

## Materials and methods

**Fig. 1:**
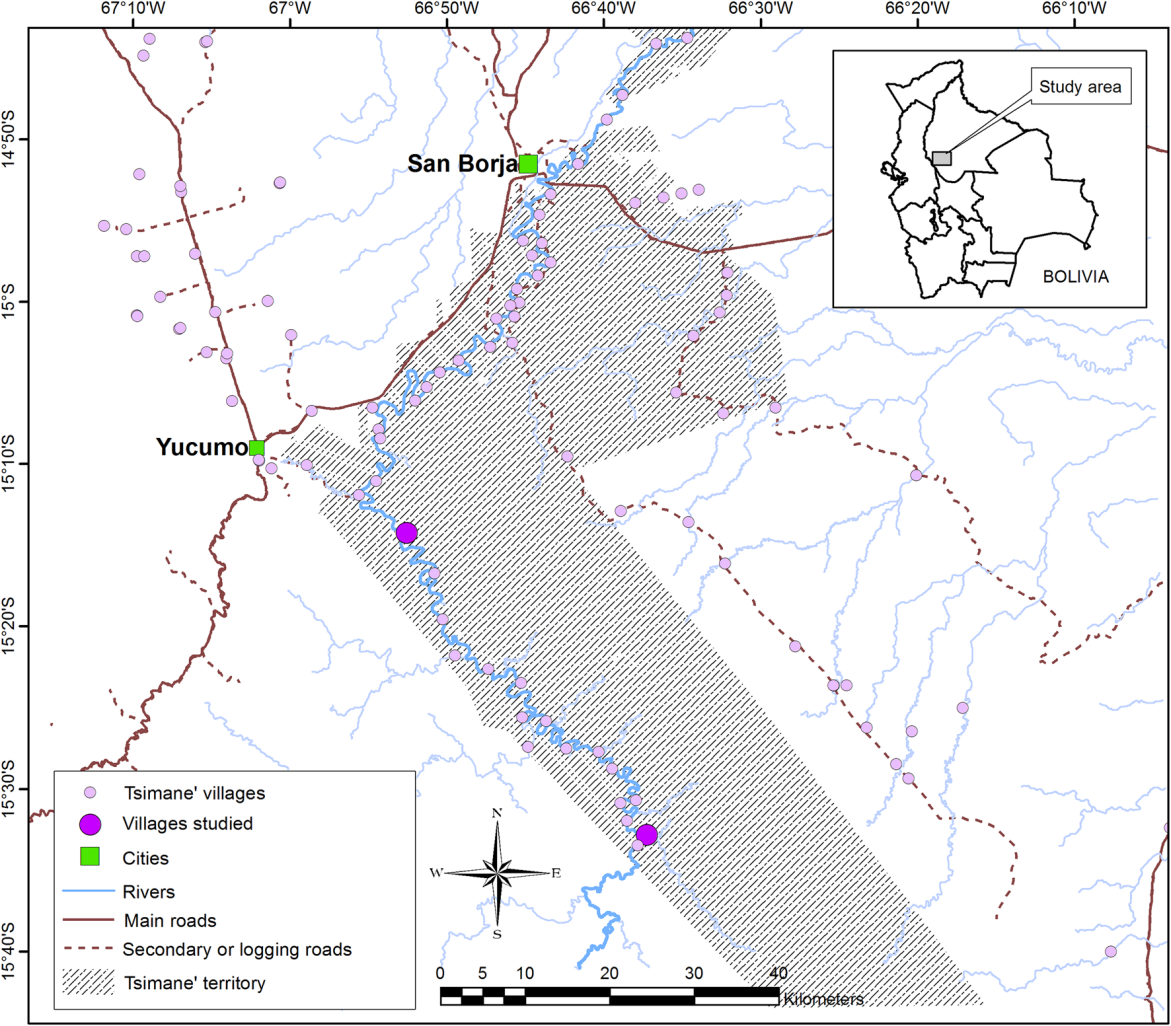
Map of the Tsimane’ Territory

We applied a mixed methods approach throughout 18 consecutive months of fieldwork. The first and second authors lived in two Tsimane’ villages from January 2012 to August 2013. We obtained consent to carry out the research from the *Gran Consejo Tsimane’* (the Tsimane’s governing body). Subsequently, a community meeting was held in two villages (V1 and V2) along the Maniqui River (Fig. 1) to explain the project to villagers and to assess their willingness to participate. Once we obtained consent to live in the villages, all adults (16 years of age and older) were invited to participate in the research. Women and men were equally encouraged to participate, resulting in a sample of 68 women and 79 men, for a participation rate above 90 % in both villages. To minimize the risks of gender bias in data collection, we trained a team of male and female assistants and translators (see Morgen 1989 in Pfeiffer and Butz 2005).

### Qualitative methods

Qualitative data were collected through participant observation in medicinal plant foraging expeditions and remedy preparation. In the first months of fieldwork, 12 semi-structured interviews were conducted with key informants (six women and six men) on the Tsimane’ healing system. In these interviews, we asked about the most common health concerns in the area and how informants responded to those ailments. To address perceptions and socio-cultural norms around access to and control over medicinal plants, biomedicines, gendered behaviours and responsibilities regarding health care giving, three focus group discussions were carried out. We invited women and men of different ages in each village to the focus groups, what resulted in two groups of ten and four women, respectively, and one group of seven men. Qualitative information was used to contextualize the research and interpret the results.

### Quantitative methods

#### Assessing the diversity of medicinal plant knowledge

The first step in estimating knowledge diversity was to identify plants with medicinal uses known by people in the two communities. To achieve a representation of knowledge held by women and men of different ages, in each study village we purposely interviewed a sample of 10 women and 10 men aged 16–91 (mean = 47 years) who were thought to hold good knowledge of medicinal plants according to the information collected through participant observation and the focus groups. We asked informants to list all of the medicinal plants they knew. We then asked the informants to list all of the ailments that could be potentially treated with each plant listed.

With results from the free-listings, we designed a medicinal plant knowledge survey consisting of structured questions about 16 medicinal plants.^1^ To develop the survey, we selected medicinal plants listed by at least two people. We calculated their salience according to each plant’s occurrence and position in the free-listings and then created three salience groups (Thompson and Zhang 2006). We randomly selected three plants from the high and low salience groups and four from the medium salience group. Women’s and men’s free-listings were analyzed separately, and six more plants (three listed only by women and three listed only by men) were selected. To administer the survey, local assistants read the vernacular names of the selected plants and asked informants whether they knew the plant and, if so, to list a maximum of three different symptoms or ailments that could be treated with it. Informants were also asked their age. A total of 63 women and 58 men answered the survey questionnaire.

We used data from the medicinal plant knowledge survey to construct the following individual-level variables: (1) number of plants known, (2) number of illnesses treated with the plants known, and (3) average number of uses per medicinal plant known. We used a Kolmogorov–Smirnov test to test for normality of distribution in our data. As data were normally distributed, we then compared the values of such variables by sex and age groups using a *t* test of mean comparisons. By aggregating women’s and men’s medicinal plant knowledge, we can explore whether these two groups hold diverse types of knowledge that may affect knowledge system performance.

#### Exploring the functional redundancy of medicinal plant knowledge

To explore functional redundancy in Tsimane’ medicinal plant knowledge, we collected data on the use of medicinal plants through a health survey. We differentiate between knowledge and use of medicinal plants because previous research indicates that the two do not necessarily overlap and medicinal plant knowledge may not be an accurate proxy for the actual use of medicinal plants and vice versa (Reyes-García et al. 2005; Wayland and Walker 2014).

The health survey was administered up to six times over the course of 12 months to the sample of 68 women and 79 men, most of whom had also answered the knowledge survey. We asked about the ailments suffered in the 2 weeks prior to the interview and the treatments used. Adults self-reported and mothers (or the adult in charge) reported children’s health. We recorded ailments or therapeutic categories as cited by the informants so that their illness classification (or nosology) was retained. The treatments reported were then coded as (1) traditional (medicinal plants or others), (2) biomedical (pharmaceuticals), or (3) mixed (a combination of traditional and biomedical treatments).

To assess functional knowledge redundancy, we evaluated the potential correspondences between the data collected through free-listings, the knowledge survey, and the health survey. Specifically, we constructed a matrix in which rows correspond to the medicinal plants used to treat the same ailment and columns correspond to our measures of the number of plants that women and men knew to treat the same ailment, including (1) the ailments most frequently reported in the health survey, and (2) the number and names of plants that women and men listed as remedies for such ailments. As these data were not normally distributed according to the Kolmogorov–Smirnov test, we calculated Spearman correlations between frequency of reported ailments and functional knowledge redundancy reported by women and men for each ailment.

Plant voucher specimens were not collected for this study. Vernacular names given by informants were recorded and linked to botanical genera based on previous ethnobotanical research in the area (Huanca 1999; Reyes-García 2001; Ticona 2010; Guéze 2011); therefore, the decision to associate a local name with a scientific species name was made only when a single member of the genus was known to be equivalent to the vernacular name reported. Vernacular names are used throughout the text; see correspondence to botanical genera in Supplementary material.

## Results

### Diversity of medicinal plant knowledge among the Tsimane’

A total of 78 medicinal plants were registered in the free-listings (Supplementary material). Some plants were reportedly used to treat only one affliction, but others, such as *ere’*, *saute*, and *tamtac*’, were used to treat up to six different illnesses.

**Fig. 2:**
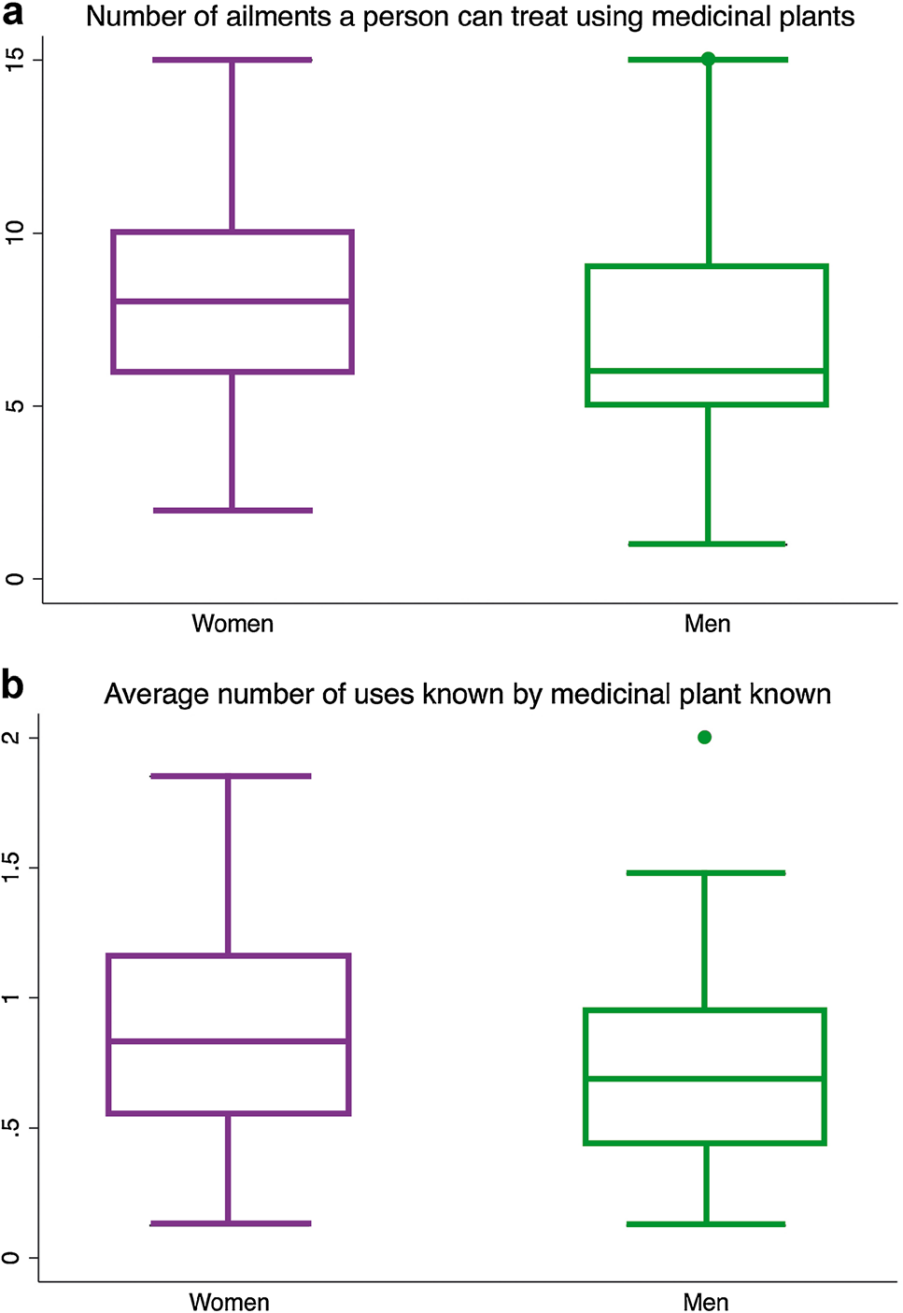
Results from knowledge survey. Comparison of box-plot distributions of **a** the number of ailments a person can treat using medicinal plants, and **b** average number of uses known by medicinal plant known

Results from the knowledge survey indicated that there is gendered variation in the ability to name medicinal uses for 16 selected plants species (or number of plants known) (*p* < 0.1) (Table 1). Women knew more medicinal uses (*p* < 0.05) and more uses per plant (*p* < 0.05) than men (Fig. 2a, b). From the various age–sex categories, young men (≤25 years of age) reported fewer medicinal plants and uses per plant than people in any other age category (*p* < 0.05), whereas older women (>50 years of age) reported the largest number of medicinal plants and uses (Table 1).

**Table 1:** Results of statistical analyses (*t* test) of women’s and men’s knowledge of medicinal plants from the knowledge survey

Variables	Definition	Total	Women (*n*)	Women	Men (*n*)	Men	*P* value
Number of plants known	No. of plants with a medicinal use (from 0 to 16)	7.61 (2.21)	63	7.95 (2.98)	58	7.25 (2.83)	0.09*
Number of different illness (uses)	No. of different illnesses treated with the selected plants	7.42 (2.95)	63	7.92 (2.93)	58	6.87 (2.90)	0.02**
Average number of uses	Average no. of uses known per medicinal plant recognized	0.81 (0.39)	63	0.87 (0.40)	58	0.74 (0.38)	0.03**
By age groups							
Number of plants known							
<25		6.26 (2.13)	19	6.78 (2.27)	15	5.6 (1.80)	0.05*
>25 < 50		8.12 (2.87)	33	8.39 (2.88)	32	7.84 (2.87)	0.22
>50		8.23 (3.50)	11	8.64 (3.93)	11	7.82 (3.16)	0.30
Number of different illness (uses)							
<25		6.35 (2.37)	19	6.89 (2.40)	15	5.67 (2.22)	0.07
>25 < 50		7.94 (3.09)	33	8.24 (3.16)	32	7.63 (3.03)	0.21
>50		7.55 (3.04)	11	8.73 (2.80)	11	6.36 (2.91)	0.03**
Average number of uses							
<25		0.65 (0.36)	19	0.74 (0.39)	15	0.54 (0.28)	0.05**
>25 < 50		0.86 (0.40)	33	0.89 (0.41)	32	0.83 (0.40)	0.26
>50		0.90 (0.38)	11	1.03 (0.36)	11	0.78 (0.37)	0.06

A close examination of the knowledge survey data suggests that gendered differences refer both to the number of plants known, to uses, and to the content of knowledge itself. For example, although women and men generally agreed on the most commonly reported medicinal uses, only women reported uses related to reproductive health (e.g., fertility, labor, and contraception) and to childhood ailments such as chickenpox. In turn, only men reported medicinal uses related to the gallbladder (Table 2).

**Table 2:** Women and men answers to the knowledge survey by medicinal plant

Tsimane’ name	Scientific name	Women				Men			
		No. of ♀ that give a medicinal use	No. of ailments that can be treated	Most common ailments that can be treated with the plant	Specific ailments that can be treated with the plant cited by ♀	No. of ♂ that give a medicinal use	No. of ailments that can be treated	Most common ailments that can be treated with the plant	Specific ailments that can be treated with the plant cited by ♂
Vambason	*Aspidosperma* aff. *rigidum* Rusby	44	15	Stomach pain, diarrhea	Menstruation	44	16	Diarrhea Back pain	Gallbladder
Macha	*Amburana cearensis*	25	13	Back pain Flu	Post labor	25	12	Back pain Muscle ache	Scabies
Buisi	*Entada* sp.	57	15	Stomach ache Menstruation	Muscle ache Contraception	40	14	Stomach pain	
Parta	*Persea americana* C. Miller	6	5	Diarrhea Back pain		10	7	Diarrhea Stomach pain	Flu
Cravu		24	11	Flu Diarrhea	Labor	26	10	Flu Tooth ache	
Jamo’tarara	*Margaritaria nobilis* L.F.	22	8	Leishmaniasis fungus	Fertility Muscle ache	17	5	Leishmaniasis Fungus	
Punuvacdyes		24	11	Infected sore (boil) Muscle ache	Muscle ache	19	10	Infected sore (boil) Muscle ache	
Que’tsejtse	*Davilla nitida* (Vahl) Kubitzki	18	5	Muscle ache		15	8	Muscle ache	
Tyi’mujmure	*Piper peltatum* Ruiz & Pav	37	12	Infected sore (boil) Fever	Fever Diarrhea	30	13	Infected sore (boil) Fever	
Yavitus		20	13	Muscle ache Dizziness	Menstruation	12	10	Muscle ache Infected sore (boil)	
Arara’	*Urera laciniata* (Goudot) Wedd urticaria.	51	8	Fungus Muscle ache		52	9	Fungus Muscle ache	
Banana		23	8	Bullet ant sting Muscle ache	Stomach pain	15	7	Bullet ant sting Muscle ache	
Ñetas		30	12	Muscle ache	Chickenpox To gain weight	20	14	Stomach pain	Menstruation
Marva		58	19	Wasp sting Labor	Menstruation Scabies pre-labor	41	14	Wasp sting Cuts	
Mature	*Acmella oleracea*	55	17	Fungus Labor	Babies weeping	48	19	Fungus Tooth ache	
Tson’sonty	*Ampelocera edentula* Kuhlm.	26	12	Leishmaniasis Fungus		33	9	Leishmaniasis Fungus	

### Functional knowledge redundancy

A total of 22 ailments were reported by different people in the health survey (Table 3, column A). The most commonly reported ailments were colds, coughs, diarrhea, body or muscle aches, and fever (Table 3, column B). When asked about the treatments used, respondents reported not using anything in 24 % of the cases, and traditional medicine use in 34 % of the cases. Women used pharmaceuticals for 29 % of the ailments they reported, whereas men used pharmaceuticals for 20 % of the reported ailments. Women used a combination of traditional and biomedicines in 13 % of the treatments, versus 20 % in the case of men. When parents were asked about the treatments administered to children, they reported not applying any treatment in 18 % of the cases, using traditional medicine in 26 %, pharmaceuticals in 39 %, and a combination of both in 16 %. Biomedicines used included pills for pain and fever relief (e.g., aspirin, diclofenac, or paracetamol), vitamins, and antibiotics.

**Table 3:** Information on ailments reported in the health survey (columns A and B), and the knowledge of medicinal plants reported that could be used to treat them in free-listings and knowledge survey (columns C–H). Each row corresponds to the set of medicinal plants reported by ailment, where Columns C, E, G are plants reported by women and D, F, H are the plants reported by men. Columns C–H are used as indicators of functional knowledge redundancy

A	B	C	D	E	F	G	H
Ailments	Report frequency	No. of plants reported by **♀** in FL as treatment	No. of plants reported by ♂ in FL as treatment	Most frequently reported plants by **♀** in FL as treatment	Most frequently reported plants by ♂ in FL as treatment	Plants reported exclusively by **♀**	Plants reported exclusively by ♂
Cold	211	18	16	ere’, shepi	shepi, chorecho	coti, cravu	ashashaj
Fever	62	2	3	shepi	bejqui, viyucure		bejqui viyucure
Cough	41	6	7	tamtac, saute	tamtac, saute		shepi’is
Backpain	33	3	3	vambason	macha	vambason	macha
Diarrhea	32	16	16	oveto’, vambason	oveto’, chura’		
Headache	30	7	2	saute, shepi, rovocdyes, viyujcure, vujnare	chito’, ufjare	saute,shepi, rovocdyes, viyujcure, vujnare	
Body ache	27	17	17	tamtac, rovocdyes, punuvacdyes	morifi, saute	vambason	arara’, mashaty, potona
Stomach pain	16	8	10		tamtac, oveto’	tamtac, saute	ujfare, vijsi
Fungus	11	9	12	tamtac, saute, conojfoto	saute, tamtac		jämecatidye, undye, uruuru
Chickenpox	11	–	–				
Anemia	9	–	–				
Vomit	5	–	–				
Skin	4	1	1	tyi’	tyi’		
Bewitchment	4	2	2	conojfoto, yän	conojfoto, yän		
Intestinal parasites	3	1	1	titij	titij		
Eye complaint	3	1	1	cajin’si	cajin’si		
Toothache	3	–	–				
Boil/skin sore	2	1	2	titij	mojmosh		mojmosh
Injury	2	2	4	yantes	Ijmeme, itsi	shiveñi	yantes
Knee pain	2	–	–				
Post labor	2	–	–				
Hemorrhage	2	–	–				

With some exceptions, we observe greater functional knowledge redundancy among all participants for the most frequently reported ailments—e.g., colds, diarrhea, and aches (Table 3, columns A–D). Overall, we found a correlation between the frequency with which an ailment was reported and the functional knowledge redundancy of medicinal plants used to treat them (women’s Spearman *ρ* = 0.68, *p* < 0.001; men’s Spearman *ρ* = 0.61, *p* < 0.05). The number of plants known per ailment was similar for women and men for all ailments except for headaches, where women knew more plants than men. Despite similar number of plants listed per ailment, there were differences in the medicinal plants that women and men reported (see Table 3, columns E–H). For example, both women and men reported 17 plants for treating body aches, but women listed *tam tac’*, *rovodyes*, and *punucvadyes* more frequently, whereas men listed *saute* and *morifi* more frequently; only women reported *vambason* and *arara’*, whereas only men reported *mashaty* and *potona*. Fever and back pain (sometimes described as body pain) presented exceptions because, although they were highly reported, knowledge related to them displayed *low* redundancy. For example, while fever was the second most frequently reported ailment, only three plants were free-listed as remedies, although three more plants were reported in the health survey. Women and men agreed more about the least frequently reported ailments (bewitchment, eye ailments, intestinal parasites, and skin conditions) both in terms of the total number and the names of medicinal plants used to treat them.

## Discussion

### Gendered knowledge as a source of knowledge diversity

Previous work suggests that Tsimane’ botanical knowledge is widely shared (Reyes-García et al. 2003), but the data presented here show that there are also gendered differences in medicinal plant knowledge. This diversity seems to reside in women’s greater knowledge of medicinal uses associated with reproductive and childhood ailments, a finding that accords with other research among the Tsimane’ showing a positive association between maternal botanical knowledge and children’s health (McDade et al. 2007). Gendered knowledge is thus a source of knowledge diversity—diverse types of knowledge are applied by different social actors to treat different ailments.

A few researchers have considered the implications of intra-cultural knowledge distribution for the resilience and adaptive capacity of knowledge systems. Some argue that a normal distribution (from a probabilistic perspective) of knowledge in a local medical system may reduce the system’s vulnerability to disturbances (Ferreira Júnior et al. 2013; Santoro et al. 2015). For example, if only one person holds specialist knowledge, when this person leaves the system the knowledge is lost (unless, of course, it has already been fully transmitted). But, if knowledge is more widely distributed, then it is less likely to disappear since there is at least partial redundancy between the bodies of knowledge held by different people. It has also been claimed that knowledge diversity may contribute to adaptability in the long-term (Ruelle and Kassam 2011). For example, in the context of climate change, a diversity of knowledge about species and their phenology may offer options for coping with variation in seasonality, plant extinctions, and local extinction of certain species.

**Fig. 3:**
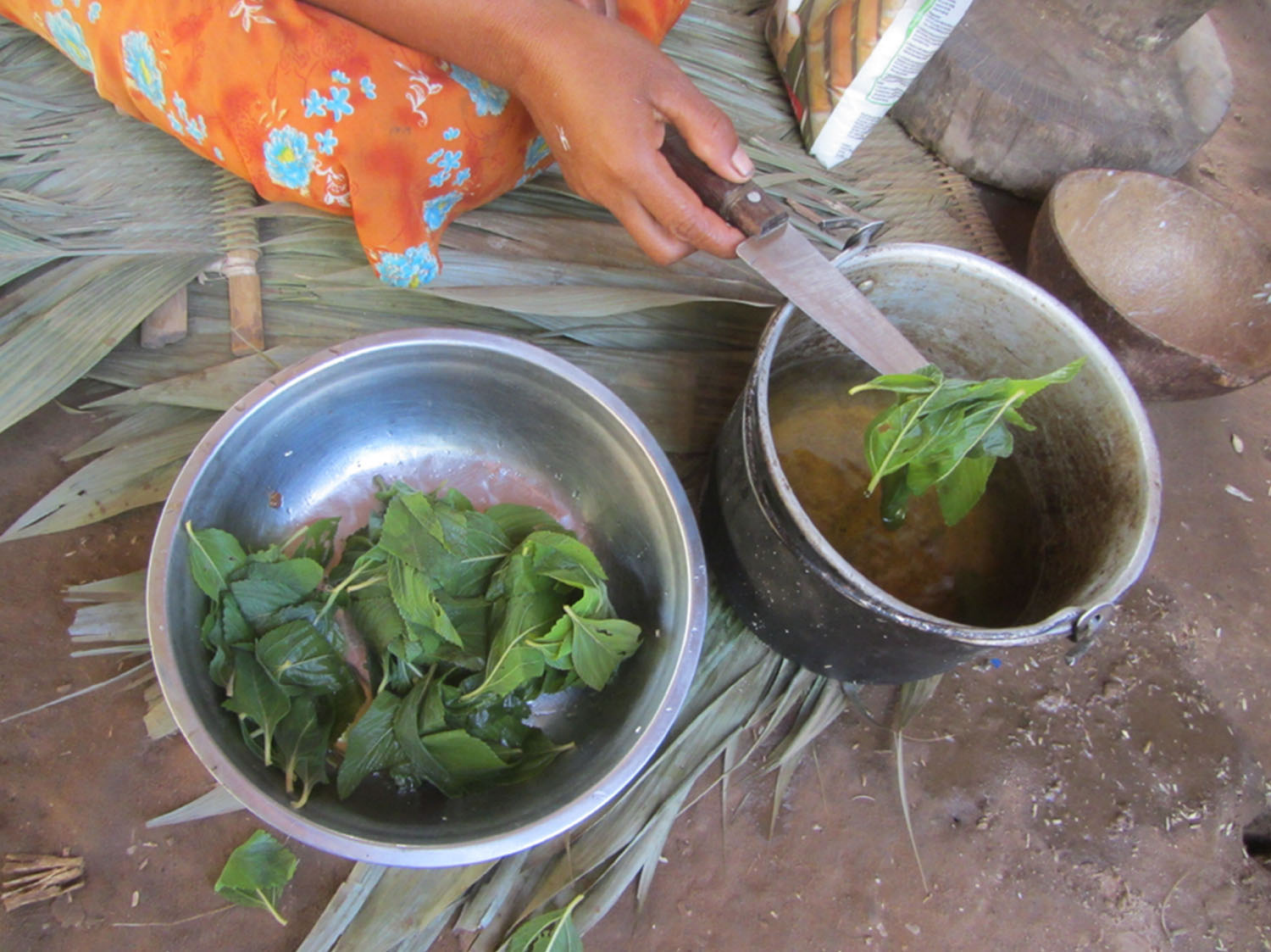
Tsimane’ woman preparing a medicine from medicinal plants *Photo* Álvaro Fernández-Llamazares

Here we have found that gender relations foster knowledge diversity which likely contributes to Tsimane’ wellbeing and adaptive capacity. Such a finding is also acknowledged by the Tsimane’ who, in focus group discussions, attributed caregiving to women (Fig. 3). As Tsimane’ women reported:

“We are the ones who take care of the family. When our children are sick we take care. We sometimes gather medicinal plants, and sometimes we ask our men to bring them; they also know where to find them” (Women, 28–78, V1, April 2012).

As principal caregivers and health custodians at household and community level, Tsimane’ women contribute with their more diverse knowledge about reproductive and childhood ailments, producing benefits that are enjoyed by the entire Tsimane’ society.

### Functional knowledge redundancy and adaptive capacity

The second important finding is that, overall, there is a greater medicinal plant functional knowledge redundancy for the most frequently reported ailments, which may offer a variety of options for treating such ailments. This is similar to what has been found in other settings. For example, the study by Santoro et al. (2015) among rural communities in northeastern Brazil showed that an ailment’s frequency of occurrence is positively associated with the number of taxa that were known to treat it. However, we also found a few exceptions, such as fever. It may be that, as a very specific symptom, it is treated with fewer plants in comparison with ailments such as colds, which may include a diverse topology of symptoms. We also observed that fever is commonly treated with pharmaceuticals. The use of an alternative treatment could, therefore, explain lower plant knowledge redundancy for this ailment.

Despite finding similar degrees of functional knowledge redundancy in the number of medicinal plants that women and men reported for frequent complaints, gendered knowledge and preferences are also evident. Differences in the plants that women and men reported contribute to redundancy within the knowledge system since, despite the fact that they reported a similar number of plants, the plants reported were different.

For some of the least frequently reported ailments, there is greater agreement between the sexes. For example, women and men reported the same plants used to treat eye ailments, intestinal parasites, and skin conditions, which may indicate that knowledge related to these ailments is widely shared (common) or that, when only a single or a few species are effective, this is more generally known. The lower functional knowledge redundancy regarding medicinal plants used to treat bewitchment, might also suggest that such treatments are more often part of the shamanic or specialized domain of knowledge.

Finally, our study also indicates that, at a practical level, Tsimane’ women and men use plant treatments slightly more often than biomedical or combined treatments. This is important, since indigenous and rural communities rely on a variety of treatment options at a local level (Giovaninni et al. 2011), and the diverse knowledge associated with them also contributes to health sovereignty (Kassam et al. 2010), as it enables individuals to opt for those treatments that they consider appropriate. As an informant reported in a semi-structured interview:

“When my son catches a cold I use medicinal plants; also to treat his scabies. If we feel very weak we can also go to the hospital. There, doctors can cure leishmaniasis but they cannot cure us if we got bewitched in the forest. Then we have to go to the *cocojsi*” (Woman, 44, V2, May 2012).

Consequently, the adaptive capacity of communities’ local knowledge and medical systems may lie in the use of medicinal plants as the primary source of healing or as an alternative to, or in combination with, biomedical options at a practical level.

## Conclusion

The conceptual framework and case study provided in this paper to assess knowledge diversity and functional knowledge redundancy within local medicinal knowledge systems may be useful for researchers who seek to evaluate adaptive capacity and resilience.

Beyond medicinal plant functional knowledge redundancy and its effects on knowledge system adaptive capacity and resilience, future research should also pay attention to complexity in the treatments, e.g., the use of combinations of medicinal plants, and of medicinal plants used together with other traditional remedies, to treat illness. The data collected to evaluate functional knowledge redundancy only considered the use of individual plants while, in practice, the Tsimane’ employ mixed remedies; thus, functional knowledge redundancy may have been underestimated. Furthermore, as adaptation to GEC emerges out of heterogeneous processes, it would be helpful to carry out research that focuses not only on the content of knowledge, but also on the context of its production, capturing the recursive relationship between knowledge and agency as mediated by power, culture, and history within adaptive dynamics (Leach 2008; Cote and Nightingale 2011). This would allow for a more nuanced understanding of the local medicinal knowledge system’s adaptive capacity and resilience.

## Electronic supplementary material

Below is the link to the electronic supplementary material.

Supplementary material 1 (PDF 70 kb)

## Footnotes

1 Available at: http://icta.uab.cat/Etnoecologia/Docs/[423]-lektests.pdf.
