# Supplementary material for: Gendered medicinal plant knowledge contributions to adaptive capacity and health sovereignty in Amazonia
Source: Ambio. 2016 Nov 22;45(Suppl 3):263–75. doi: 10.1007/s13280-016-0826-1 (PMC5120019; doi:10.1007/s13280-016-0826-1)
Supplement: Supplementary file 1 — Supplementary material 1 (PDF 70 kb) [file 13280_2016_826_MOESM1_ESM.pdf]

Ambio

Electronic Supplementary Material

This supplementary material has not been peer reviewed.

Title: **Gendered medicinal plant knowledge contributions to adaptive capacity and health sovereignty in Amazonia**

Authors: Díaz-Reviriego, I. Fernández-Llamazares, Á., Salpeteur, M. Howard, P.L, Reyes-García, V.

Supplementary material. Correspondence of vernacular with scientific names

Vernacular name	Scientific name	Taxonomic family
apainiqui	*Pera benensis* (Rusby)	Euphorbiaceae
arara	*Urera laciniata* (Goudot) Wedd *urticaria*	Urticaceae
ashashaj	*Citrus limon* (L.) Burn	Rutaceae
Ava' ava'	*Prockia crucis* L.	Salicaceae
banana	*Musa x acuminata*	Musaceae
bätin	*Syagrus sancona* Karsten	Palmae
bejqui	*Hymenaea courbaril* L.	Leguminosae-Cae
buisi	*Entada sp.*	Leguminosae-Mim
caji'si	*Martinella ovovata* (H.B.K) Bureau & Schumann	Bignoniaceae
cam	*Otoba parvifolia* (Markgraf) A. Gentry	Myristicaceae
canau		
cashcaria	*Cinchona* cf. *Officinalis* L.	Rubiaceae
cata		
ca' babas	*Olyra latifolia*	Gramineae
chij	*Triplaris americana* L.	Polygonaceae
chito	*Tephrosia vogelii J. D.* Leguminosae-Pap	Leguminosae-Pap

chorecho’	*Aniba canelilla* (H.B.K.) Mez	Lauraceae
chujbubuty	Peperomia rotundifolia (L.) Kunth	Piperaceae
chura’	*Swietenia macrophylla* (King)	Meliaceae
conojfoto	*Hura crepitans* L.	Euphorbiaceae
copaiva	*Copaifera reticulata* Ducke	Fabaceae
cos	*Nicotina tabacum*	Solanaceae
coti’	*Psidium guajava L.*	Myrtaceae
cravu		
curi	*Lantana cf. Aristat*	Verbenaceae
curu		
dyesatdyes	*Baccharis trinervis* (Lam) Pers.	Compositae
ere’	*Petiveria alliacea* L.	Piperaceae
ibiñe		
ijmeme	*Myrcia fallax*	Myrtaceae-Leg
irepij	*Ocimum micranthum* Willd.	Labiatae
itsi	*Picramnia* aff. *Sellowii* Planchan subsp *sprucanea* (Engl.) Pirari	Simaroubaceae
jamo’tarara	*Margarita nobilis* L.F	Euphorbiaceae
macha	*Amburana caerensis*	Fabaceae
manai	*Attalea phalerata C.* Martius ex Sprengel	Palmae

marva	*Sida rhombifolia L*	Malvaceae
maschaty		
mature	*Acmella oleracea*	Compositae
merique	*Ananas comosus*	Bromeliaceae
mojmosh		
morifi	*Dichorisandra sp.*	Commelinaceae
nashdyes		
orotas		
oteti		Amarilliadaceae
oveto	*Uncaria guianensis (Aubl.)*	Rubiaceae
oyoj' oyoj	*Urvillea sp.*	Sapindaceae
parta	*Persea americana* C. Miller	Laureaceae
potona	*Kalanchoe pinnata* (Lamark) Persoon	Crasulaceae
punuvacdyes		
que'tsetsej	*Davilla nitida* (Vahl) Kubitzki	Dilleniaceae
rovocdyes		
saute	*Zingiber officinale*	Zingiberaceae
sebiria	*Cymbopogon citratus*	Gramineae
shepi	*Gallesia integrifolia* (Sprengel) Harms	Phytolaccaceae
shepi'is	*Mansoa alliacea* (Lamark) A. Gentry	Bignoniaceae
shiveñi		

siyamo	*Cedrela odorata* L.	Meliaceae
sicoco	*Chenopodium ambrosioides*	Chenopodiaceae
tamtac	*Galipea longiflora* K. Krause	Rutaceae
tiribui		
titij	*Ficus insipida* Willd	Moraceae
tson' sonty	*Ampelocera edentula* Kuhml	Ulmaceae
tubuij	Gouania adenophora	Rhamnaceae
tyi'	*Genipa americana* L.	Rubiaceae
tyi'mujmure	Piper pelatum Ruiz&Pay	Piperaceae
ufajre	*Brugmansia arborea* (L.)	Solanaceae
undye		
u'puyu	Piper laevigatum Kunth	Piperaceae
vambason	*Aspidosperma rigidum*	Apocinaceae
vashi	*Serjania caracasana*	Sapindaceae
vayori	*Sparattanthelium glabrum*	Hernandiaceae
vijsi		
viyucure		
vujnare		
yäcäni		
yän		
yantes		
yavitus		

References consulted for linking vernacular names with scientific names

Guéze, M. 2011. Evaluation of tree diversity and utilization: the role of acculturation. A case studyin the Bolivian Amazaon. PhD Thesis, Institut de Ciéncia i Tecnologia Ambientals, Universitat Autònoma de Barcelona.

Huanca, T. 1999.Tsimane' Indigenous Knowledge. Swidden Fallow Management andConservation. PhD Thesis, University of Florida

Reyes-García, V. 2001. Indigenous people, ethnobotanical knowledge, and market economy. A case study of the Tsimane' Amerindians in lowland Bolivia. PhD Thesis, University of Florida

Ticona, J.P. 2010. Los chimane: conocimiento y uso de plantas medicinales en la comunidad de Tacuaral del Matos, Provicia Ballivian, Departamento del Beni. Bachelor Thesis, Universidad Mayor de San Andrés.
